# Neuralgia Faciei

**Published:** 1859-08

**Authors:** Samuel A. Parker

**Affiliations:** Dentist to the Birmingham Dental Dispensary


### NEURALGIA FACIEI.

Communicated by Samuel A. Parker, Dentist to the Birmingham Dental Dispensary,

A case came under my notice some short time ago, the patient suffering from neuralgic pains of the face.

She consulted me respecting some artificial teeth, to fill up a considerable gap in the upper jaw.

Thinking the case instructive, and of some importance, I beg to transmit the particulars respecting it for publication in the pages of the *Quarterly Journal of Dental Science*:—

Jane ——, about twenty-six years of age, was sent to me for the purpose of having some artificial teeth made for the upper jaw.

Upon examination, I found a great number of stumps remaining, the gums painful and much inflamed, and the patient subject to violent pains in the head and temples, the pain at times being so intense that no rest could be obtained, day or night. She had been in the habit of taking a considerable quantity of medicine for two years to allay the pain, but without the slightest benefit.

My course of action was at once decided, and I recommended the immediate removal of all the stumps, as the only remedy, for a speedy cure.

To this she hesitated for some time; but at length consented to have two out to begin with; which two I removed on the 11th of September last. Two more were removed on the 14th and 16th, three on the 18th, and two on the 20th of the same month, making altogether eleven stumps.

On the 16th, she told me the pain was decidedly better, had taken no more medicine, and had passed good nights.

By the 20th the pain had entirely subsided, the gums had begun to resume their healthy appearance, and the patient altogether quite recovered. In about two months afterwards she came, requesting the artificial teeth, being very impatient to have them, on account of the gap made by the removal of the stumps. I took the model, and by the 24th of November fixed them in, she finding them, in a few days, a source of great comfort, both for mastication and articulation.

This is one of those cases liable to come frequently under the dentist's care, but more frequently under the care of the medical practitioner. The case just alluded to was under medical treatment for more than two years, and yet in a fortnight after the removal of the stumps a perfect cure was established. It is a well known fact, that until every remedy (excepting the right one) has been tried, the teeth are not suspected of being the cause of such pains.

The dentist is generally consulted as a last resource, when in point of fact he should have been the first.

I cannot conclude these remarks without quoting a few words from an admirable paper by Dr. Hardy, in the *American Journal of Dental Science*, for October, 1849. The paper related to the "Pathology of the Mouth," and in the sixth page he says—"It is known to every well-informed physician and dentist, that cases have occurred, and are recorded, where patients have, year after year, taken medicine of every kind, and from the hands of the greatest variety of physicians, and all without avail; that such patients have, further, by the advice of their medical attendants, gone in pursuit of health, and for the removal of their diseases, pronounced to to be beyond the reach of medical and surgical skill; have gone by the best medical advice for the cure of their several cases, to distant regions, crossing seas, scaling mountains, visiting springs, taking every variety of exercise, and every variety of diet, yet all of no avail—as they have severally returned home, with little or no improvement in their disease, which still exhibits the same character and unmanageableness as when they started on their tour.

"By some lucky or accidental suggestion, the *teeth* are suspected as the probable cause of the whole mischief, and with some degree of plausibility, as everything else had failed to give any relief.

"This suggestion is acted upon; skillful dentists are employed, and by correcting the diseased condition of the teeth and mouth, the before-incurable diseases often become readily curable, the general health speedily restored, and the whole of this wonderful change brought about, simply by the application of a little dental skill."—*Ib.*

